# Implementing Best Practice When Screening Birthing People for a Substance Use Disorder

**DOI:** 10.1111/jmwh.13697

**Published:** 2024-10-23

**Authors:** Sheila Kaufman, Patricia D. Suplee, Damali M. Campbell‐Oparaji, Julie Blumenfeld

**Affiliations:** ^1^ Virtua Health Camden New Jersey; ^2^ School of Nursing‐Camden, Rutgers, The State University of New Jersey Camden New Jersey; ^3^ Department of Obstetrics and Gynecology Rutgers New Jersey Medical School Newark New Jersey; ^4^ Midwifery Program, Division of Advanced Nursing Practice School of Nursing, Rutgers, The State University of New Jersey Newark New Jersey

**Keywords:** certified nurse‐midwife, certified midwife, midwifery, prenatal care, racism, substance‐related disorders, substance abuse detection, toxicology

## Abstract

Screening for substance use disorder (SUD) is an essential part of antepartum care. Best practice for screening requires the use of a validated tool early in pregnancy to identify those at risk and to connect them with counseling and treatment. In many health systems and practices, urine toxicology testing is erroneously employed as a SUD screening tool despite consistent recommendations against its routine use. The results are often misinterpreted as diagnostic of SUD and can have harmful downstream effects for pregnant and birthing people. This Clinical Rounds reviews the tools available for evidence‐based SUD screenings in pregnancy care, pitfalls of urine toxicology testing, and ways in which midwifery care is well‐positioned to implement evidence‐based screening practices in pregnancy care.

## CASE SUMMARY

*M.U. (she/her) is a 28‐year‐old, gravida 3 para 2 who presented for antepartum care at 10 weeks’ gestation. At M.U.’s initial visit, the 4P's Plus verbal screening tool was used to assess for substance use disorder (SUD). M.U. disclosed past cannabis for recreational use before pregnancy and reported no other substances. At 14 weeks, M.U. returned and disclosed to the midwife that she had been taking oxycodone and acetaminophen (Percocet) for chronic back pain but tried to self‐wean by obtaining varied amounts of buprenorphine and naloxone (Suboxone) in the community. M.U. was no longer was using Percocet but felt that the Suboxone was not meeting her needs and she was experiencing withdrawal symptoms*.

*M.U. went on to share that her previous experience with urine drug testing had made her hesitant to disclose her current use at the first visit. She reported that in her prior pregnancy, during admission for labor, a urine drug test was performed without her consent or knowledge. She was told that the test came back positive for the presence of Phencyclidine (PCP), which M.U. insisted she was not using. While awaiting confirmatory testing, M.U.’s newborn was removed from her care by child protection and welfare services. After 5 days, confirmatory testing showed no presence of PCP, and M.U.’s newborn was returned to her. M.U. reported that she had difficulty breastfeeding due to the separation. M.U. shared now that her goal was to have a negative urine drug test on admission to the hospital and requested assistance with meeting this goal. The midwife thanked M.U. for sharing this information and affirmed her feelings of fear about disclosing this information. The midwife proceeded to discuss how substance use can affect M.U. and her fetus* *, the benefits of SUD treatment in pregnancy, and the availability of programs in the community to support pregnant people in need of treatment. The midwife also reviewed local state regulations on mandated reporting of SUD in pregnancy and childbirth. M.U. was connected with a medication for opioid use disorder (MOUD) program for pregnant individuals, where she was prescribed Suboxone and received counseling. M.U. went on to attend both MOUD and prenatal care appointments throughout the pregnancy. During her MOUD visits, she consented to routine urine drug testing, which demonstrated adherence to the program. M.U. entered spontaneous labor at 38 weeks and gave birth to a healthy newborn and was able to successfully breastfeed. In accordance with local laws, M.U.’s case was referred to child protection and welfare services for review. After confirmation of her participation in the MOUD program, her case was closed at 6 weeks postpartum without incident*.

Continuing education (CE) is available for this article. To obtain CE online, please visit http://www.jmwhce.org. A CE form that includes the test questions is available in the print edition of this issue.

*Note: This case is a composite of elements from several patients*.

## INTRODUCTION

Leading perinatal health professional organizations, including the American College of Nurse‐Midwives, the American College of Obstetricians and Gynecologists, the Society for Maternal‐Fetal Medicine, and the Substance Abuse and Mental Health Services Administration, recommend universal screening for SUD early in pregnancy using a validated screening tool.^1^, ^2^, ^3^ However, practices and health care systems continue to use urine drug testing, universally or selectively, in lieu of screening.^4^ Urine drug tests detect the presence or absence of specific drugs or their metabolites in a person's body at one point in time, and results may be falsely positive and falsely negative.^5^ Despite public policy messaging to the contrary, drug tests do not diagnose a SUD, nor are they indicators of individual capacity to parent. The intended purpose of both SUD screening and testing should be to guide recovery and not to inflict punitive measures.

## RISKS AND SEQUELAE OF SUD IN PREGNANCY

SUD is common in the United States and in pregnant people, rendering evidence‐based screening for SUD a public health imperative. Without treatment, SUD may have deleterious consequences including mortality for pregnant people and neonatal opioid withdrawal syndrome for neonates.^2^, ^3^ According to recent data, the rate of maternal opioid use in the United States is 8.2 per 1000 birth hospitalizations, and the rate of neonatal abstinence syndrome is 7.3 per 1000 birth hospitalizations.^6^ An increasing number of individuals in the United States die secondary to drug overdose. According to the most recent available data from the Centers for Disease Control and Prevention, in 2021 there were 106,699 deaths in the general population due to drug overdose.^7^ The rate of these deaths increased 14% from 2020 to 2021.^7^ Between 2017 and 2019, data from US maternal mortality review committees reported that mental health conditions, which included overdose‐related deaths, represented the most common underlying cause of pregnancy‐related deaths.^8^ Furthermore, substance use was determined to be a contributing factor in a quarter of all pregnancy‐related maternal deaths.^8^ The rising number of overdose deaths in the pregnant population is alarming. This is exemplified in recent data from Maryland, Texas, and Alaska where, respectively, 15%, 17%, and 22% of pregnancy‐associated deaths were due to substance use.^9^, ^10^, ^11^ Bruzelius and Martins noted an 81% relative increase in the number of pregnancy‐associated overdose deaths from 2017 to 2020.^12^ Campbell et al reported that almost all pregnancy‐associated overdose deaths were related to opioid use with polysubstance use on the rise.^13^

Both short‐term and long‐term effects of substance intake during pregnancy on newborns vary, and it may be challenging to discern the distinct effects of substances taken concurrently. For example, opioid‐exposed neonates can experience withdrawal symptoms that are often transient, and most are successfully addressed with nonpharmacologic treatments.^14^ In contrast, alcohol, which has teratogenic effects in pregnancy, can cause long‐term, irreversible intellectual deficits.^15^ Additionally, assessing potential longitudinal outcomes of substance use is confounded by diverse factors. The health of the pregnant person and structural and social determinants of health, inclusive of racism, also affect fetal growth and development.^16^

## BIAS IN SCREENING AND TESTING

Universal screening for SUD is an evidence‐based initial intervention to appropriately identify individuals in need of support and treatment. Despite agreement among professional organizations that use of a validated SUD screening tool is best practice, no single screening tool has currently been endorsed.^17^ This leaves the decision of how to assess for SUD up to individual health care providers and systems, which can create the potential for bias and discrimination. Research has shown that perinatal health care providers and systems have relied on biases such as perceived inadequacy of prenatal care, age, and race as guiding factors in screening pregnant people.^18^, ^19^, ^20^ Studies also show that perinatal health care providers have disproportionately targeted women of color for urine drug testing, leading to inaccurate predictions as to who is using illicit drugs during pregnancy.^18^, ^19^, ^20^ For example, a 2022 retrospective chart review of SUD testing practices among 6000 birthing people found that although most individuals tested were Native Hawaiian or Pacific Islander, White individuals more often tested positive.^18^ Similarly, Jarlenski et al reported that Black patients in their study had a urine drug test ordered more often than White patients even when there was no reported history of a SUD. Furthermore, Black individuals in the study had less probability of a positive test result than other racial groups.^19^

## LIMITATIONS OF BIOLOGIC TESTING

Biologic testing, most commonly urine drug testing, produces an isolated snapshot of substance use but does not inform clinicians as to frequency, quantity, or duration of use, that is, it does not provide a diagnosis of a SUD but is too often conflated to indicate this diagnosis.^21^ The American Society of Addiction Medicine has emphasized that “equating a positive toxicology test with child abuse or neglect is scientifically inaccurate and inappropriate, and can lead to an unnecessarily punitive approach, which harms clinician‐patient trust and persons’ engagement with healthcare services.^22^ ^(p 2)^” This policy recommendation is echoed by professional midwifery and medical societies.^1^, ^2^

Urine toxicology tests vary in the type of substances screened and the sensitivity of the tests performed. Substances that can be found and measured in urine include opioids, hallucinogens, depressants, stimulants, and cannabinoids.^23^ For both efficiency of time and cost, the initial testing is an immunoassay that cannot distinguish between certain substances and therefore may generate a false‐positive result.^23^, ^24^ For example, commonly used medications in pregnancy such as labetalol, ranitidine, and even metformin can produce false‐positive results for the presence of amphetamines.^24^ False‐positive results for PCP, as in the case of M.U., can occur with the use of several over‐the‐counter medications such as doxylamine (Unisom), diphenhydramine (Benadryl), and dextromethorphan.^23^ Therefore, any positive result should be verified with chromatography of the original sample to confirm the specific substance present.^23^ Most substances are detectable for several days following use; however, some long‐acting barbiturates and benzodiazepines can persist for weeks.^23^, ^24^ Cannabinoids can remain detectable for longer periods of time when used in greater quantities.^23^, ^24^ Furthermore, lack of health care provider understanding of and ability to manage false‐positive results can have a profoundly detrimental impact on patients.^25^

Another significant drawback of urine drug testing is that it generally does not include alcohol, as it is rapidly metabolized and eliminated.^24^ Moeller et al point out that the metabolite, ethyglucuronide, can be detected for up to 5 days after intake but that exposure to hand sanitizer and mouthwash could also produce positive results.^24^ This highlights another aspect of urine drug testing in that the results are dependent on the rate of metabolism and hydration status and not the quantity ingested.^23^ Although some studies find that urine toxicology tests are slightly more sensitive for recent substance use,^26^ Bharat et al's extensive review found that self‐report assessments (a written or verbal tool) overall agreed with confirmatory urine toxicology and at a much lower cost.^27^ Additionally, screening tools are not limited by the relatively short length of time substances remain detectable in urine.

## BEST PRACTICE IN SUBSTANCE USE SCREENING

Screening all pregnant persons for substance use at the first prenatal encounter with a validated screening tool has been endorsed by professional organizations.^1^, ^2^ Available screening tools consist of a limited number of questions about current or past use of specific substances or other situations that would increase risk, such as family history of SUD. A positive screen result identifies individuals who are at increased risk for SUD and is followed by further assessment and appropriate interventions for risk reduction.

Several screening tools have been designed for or validated within pregnant populations. These include the Tobacco, Alcohol, Prescription medication and other Substance use (TAPS), the CRAFFT screening tool, and the 4 P's Plus.^2^, ^28^

**Figure 1:**
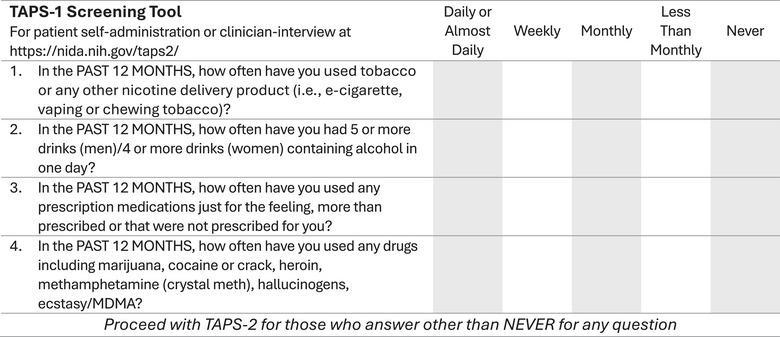
Tobacco, Alcohol, Prescription Medication, and Other Substance Use Screening Tool Abbreviation: TAPS, Tobacco, Alcohol, Prescription medication, and other Substance use. Source: Adapted from NIDA.^29^

The TAPS screening tool was developed with support by the National Institute on Drug Abuse (NIDA). This tool consists of TAPS‐1, a brief initial screen (Figure 1) typically used at the first prenatal encounter.^29^ Any affirmative answer triggers the administration of the TAPS‐2, a companion follow‐up to clarify details of substances used. TAPS‐1 and ‐2 are validated for patient self‐administration or health care provider interview. NM‐ASSIST, now known as TAPS in its electronic format, has shown moderate to high sensitivity in pregnant populations.^30^, ^31^ NIDA screening tools are freely available online as well as in electronic format. TAPS‐1 consists of 4 questions and can be completed within one minute. The total number of questions in TAPS‐2 varies, depending on the responses to individual questions.^29^

**Figure 2:**
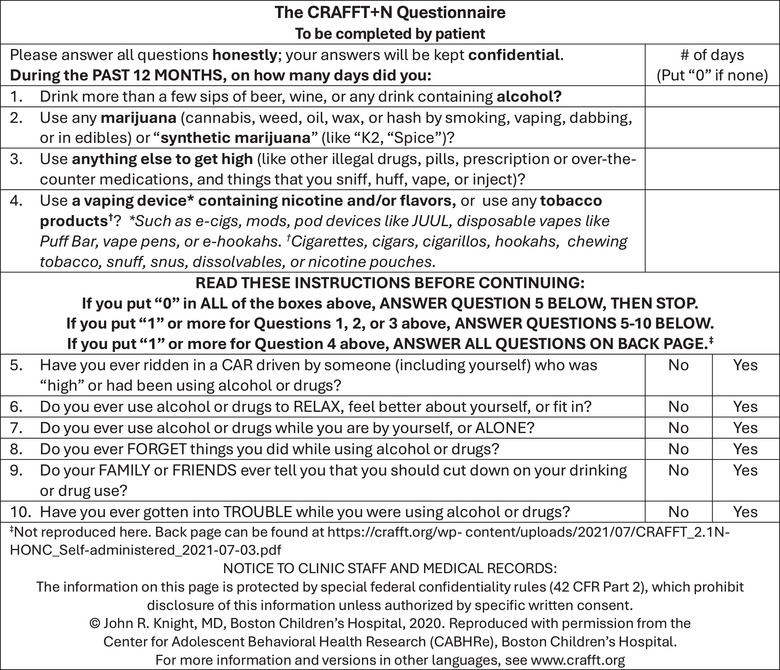
The CRAFFT Screening Questionnaire for Self‐Administration Source: Reprinted with permission from the Center for Adolescent Behavioral Health Research.^32^

The CRAFFT screening tool (Figure 2) was developed specifically for adolescent populations and has been validated in individuals aged 12 to 26 and in pregnant populations.^32^ The current version, CRAFFT 2.1+N, has been updated to include screening for cannabis, tobacco, and nicotine consumption, as well as vaping.^32^ The screening tool can be either self administered or health care provider administered.^32^ CRAFFT consists of 4 initial questions that can be completed within one minute and is freely available in more than 30 languages.

4P's Plus (Parents, Partner, Personal past, current Pregnancy) is a proprietary screening tool specifically designed for pregnant adult individuals to assess for risks of SUD.^33^ Use of 4P's Plus requires a licensing fee that starts at $2500 per year.^33^ The tool guides respondents through clarifying questions and quantification of use of substances to stratify risk of SUD. The screening includes use of tobacco, alcohol, cannabis, and illicit drugs and was validated when used as part of a verbal patient interview.^33^ It has been translated into 5 languages and consists of 5 initial questions that can be completed within one minute.^33^

## SOCIAL AND LEGAL IMPLICATIONS

Given the limited clinical picture that urine drug testing imparts, clinicians must carefully consider the negative implications of using this method for universal screening. Urine drug testing does not inform the reasons for use (eg, chronic pain), chronicity of usage, how use impacts relationships and responsibilities, or whether the pregnant person has the desire or resources to reduce or discontinue use.^21^ There are social and legal ramifications of a positive drug test result in many states that may follow a patient far beyond their pregnancy care and birth. As such, it is essential that health care providers engage in dialogue with their patients regarding potential implications of urine toxicology testing, including benefits and risks before proceeding. It is also imperative that written consent for testing is obtained.^22^ An approach such as shared decision‐making may reduce risks of coercion, encourage listening, and take patient's desires into consideration.

Evidence shows that health care providers order drug testing disproportionately for Black and Indigenous pregnant people relative to their White counterparts.^21^, ^34^ When testing is positive, providers report Black and Indigenous pregnant people more frequently to social services and child protective services (CPS).^35^ This, on the micro level, leads to devastating downstream consequences and, on the macro level, furthers existing racial inequities.^21^, ^34^, ^35^

Due to bias, SUD historically has been, and continues to be, framed as poor social or criminal behavior rather than a legitimate health condition.^21^, ^22^ Perinatal health care providers in the United States are mandated to report substance use during pregnancy in 36 states as part of data reporting or referral for evaluation or treatment.^36^ In 25 of these states, reporting of substance use to state child welfare agencies is also mandated.^36^ Although not a criminal offense, substance use in pregnancy is grounds for civil commitment (eg, court‐ordered treatment) in some states.^36^ State policies that enforce child welfare agency intervention or mandate pregnant people into state custody or treatment facilities have been shown to discourage pregnant people from seeking health care for their pregnancy and their substance use.^22^, ^37^ In the United States, suspected substance use that leads to CPS involvement may result in parent child separation and family hardships.^37^ Removal of children from their families and into the foster care system due to suspected or actual drug use has affected and continues to disproportionately affect Black, Latinx, and Indigenous children.^21^, ^37^ Despite a lack of evidence of a causal relationship between the use of drugs and child mistreatment, this practice continues in many areas.^21^, ^37^

The federal Child Abuse Prevention and Treatment Act (CAPTA) was passed in 1974 to investigate and mitigate mistreatment of children in the United States and has evolved over time largely in response to social changes and political influences.^38^ For example, in 2003 CAPTA initiated a requirement for health care providers working in states receiving federal funding to notify CPS when they cared for newborns affected by fetal alcohol syndrome or substance use.^37^ The passage of the Comprehensive Addiction and Recovery Act in 2016 further amended reporting requirements to include Plans of Safe Care. This measure was designed to ensure the safety of birthing people and their newborns and provide support for treatment.^39^ Despite its intention, this legislation and its iterations over time have resulted in many states endorsing the practice of testing newborns for exposure to drugs, reporting all exposed newborns to CPS, and creating an enduring culture that frames a positive toxicology test as child neglect or abuse.^37^ Additionally, health care providers may be faced with conflicting mandates when policies require testing or reporting that has been shown to be counterproductive and potentially harmful. This compounds a lost opportunity to respond with supportive measures.

## IMPLICATIONS FOR PRACTICE

SUD is treatable, and because of their skill set and training in relationship‐based care, midwives are ideal health care providers to engage individuals with SUD. This begins with standard incorporation of education regarding SUD screening and treatment in midwifery education programs. Additionally, professional resources are available to guide the initiation and ongoing management of SUD in pregnant people as well as opportunities to collaborate with and refer to specialists, including mental health care providers and community‐based peer support specialists.

The care provided by M.U.’s midwife demonstrates best practice and evidence‐based care. M.U. was appropriately screened with a validated tool at her initial visit and a relationship was established. At the subsequent visit, she felt safe enough to disclose her struggles with SUD and readiness to engage in treatment. M.U.’s experience in her first pregnancy demonstrates how urine drug testing, with or without consent, can lead to unintended negative consequences and perpetuate the distrust in health care systems held by so many birthing people today. For those who work in systems that require urine drug testing, it is essential that health care providers advocate for change in policies to meet the standards for best practice.

## CONCLUSION

There are a variety of screening tools for SUD endorsed by professional organizations available for use in clinical practice. Universal utilization of these screening methods requires minimal time investment while offering potential for significant risk reduction. Appropriate screening for SUD in pregnancy with validated tools is an essential strategy to mitigate the rise of pregnancy‐related deaths and the sequalae for neonates and their families.

## CONFLICT OF INTEREST

The authors have no conflicts of interest to disclose.
